# The Temporal Lagged Relationship Between Meteorological Factors and Scrub Typhus With the Distributed Lag Non-linear Model in Rural Southwest China

**DOI:** 10.3389/fpubh.2022.926641

**Published:** 2022-07-22

**Authors:** Hongxiu Liao, Jinliang Hu, Xuzheng Shan, Fan Yang, Wen Wei, Suqin Wang, Bing Guo, Yajia Lan

**Affiliations:** ^1^West China School of Public Health and West China Fourth Hospital, Sichuan University, Chengdu, China; ^2^Panzhihua City Center for Disease Control and Prevention, Panzhihua, China; ^3^Institute of Health Policy & Hospital Management, Sichuan Academy of Medical Sciences & Sichuan Provincial People's Hospital, Chinese Academy of Sciences Sichuan Translational Medicine Research Hospital, Chengdu, China

**Keywords:** meteorological factors, scrub typhus, distributed lag non-linear model, early warning, rural areas

## Abstract

**Background:**

Meteorological factors can affect the emergence of scrub typhus for a period lasting days to weeks after their occurrence. Furthermore, the relationship between meteorological factors and scrub typhus is complicated because of lagged and non-linear patterns. Investigating the lagged correlation patterns between meteorological variables and scrub typhus may promote an understanding of this association and be beneficial for preventing disease outbreaks.

**Methods:**

We extracted data on scrub typhus cases in rural areas of Panzhihua in Southwest China every week from 2008 to 2017 from the China Information System for Disease Control and Prevention. The distributed lag non-linear model (DLNM) was used to study the temporal lagged correlation between weekly meteorological factors and weekly scrub typhus.

**Results:**

There were obvious lagged associations between some weather factors (rainfall, relative humidity, and air temperature) and scrub typhus with the same overall effect trend, an inverse-U shape; moreover, different meteorological factors had different significant delayed contributions compared with reference values in many cases. In addition, at the same lag time, the relative risk increased with the increase of exposure level for all weather variables when presenting a positive association.

**Conclusions:**

The results found that different meteorological factors have different patterns and magnitudes for the lagged correlation between weather factors and scrub typhus. The lag shape and association for meteorological information is applicable for developing an early warning system for scrub typhus.

## Introduction

Scrub typhus, also known as tsutsugamushi disease, is an acute febrile illness caused by infection with *Orientia (O.) tsutsugamushi* (1). Scrub typhus is a well-known serious public health problem in the Asia-Pacific area that threatens approximately one billion people; moreover, one million people may develop illness from scrub typhus each year globally (2). During the last two decades, scrub typhus has been increasingly reported and has become a significant health concern in eastern Asian countries (3, 4).

Transmission depends on the seasonal activities of both chiggers and humans (5). First, chiggers are most abundant during rainy seasons whereas very few are found during the dry winter months (5). Second, outdoor workers, particularly field workers in rural areas, have a higher risk of acquiring the disease (5, 6). People working in farms and forestry where the chigger-infected rodent cycle occurs have a prolonged duration of exposure and are in danger of infestation with infected mites (3, 7). In China, farmers represent the most commonly infected occupation, accounting for 72.58% of all cases (3).

To date, many studies have evaluated the association between meteorological factors and vector-borne diseases around the world (7, 8) These findings have mainly provided evidence of the association between climate change and diseases. Gage et al. (9) reported that temperature, precipitation, humidity, and other climatic factors were known to affect the reproduction, development, behavior, and population dynamics of the arthropod vectors of these diseases.

Vector mite species can cause different seasonal patterns of scrub typhus as a result of different species and genotypes (2), For example, there are two seasonal peaks of the diseases in China: the summer type and the autumn-early winter type (3), which are primarily caused by the larvae of the chigger mites *Leptotrombidium deliense* and *L. scutellare*, respectively. In Southwest China, the summer seasonal peak is the most common type. Some findings have shown that the seasonality of the disease in these regions is related to the lifecycle of *L. delicense*, which is a predominant vector of Karp-type scrub typhus (8).

The effects of meteorological factors are observed to have two main aspects: lag and non-linear characteristics (10). However, research quantitatively exploring the lag association between weather variables and scrub typhus is sparse (11). Existing studies have validated the non-linear correlation between weather factors (for example, temperature and rainfall) and scrub typhus (12, 13). However, in Southwest China, until now, there has been no report on lag structures and association patterns between weather variation and scrub typhus. The study area in our investigation includes a mountain area, complex terrain, and existing distinguishable dry and rainy seasons. In fact, scrub typhus is hyperendemic in the Panzhihua District, which is located in Southwest China, indicating that its incidence is higher, specifically ~10 times higher than the average incidence in Sichuan Province overall (3); moreover, the highest incidence occurs in the countryside. Furthermore, the lag structures and association patterns between weekly meteorological variation and scrub typhus require further investigation, particularly in the countryside.

The objective of our work was to explore the lag structures and association patterns between meteorological variables and scrub typhus in rural areas of Panzhihua district, Southwest China. The distributed lag non-linear model (DLNM) was used to study the temporal lagged associations between weekly meteorological factors and weekly scrub typhus cases using data from 2008 to 2017.

## Methods

### Study Area

**Figure 1:**
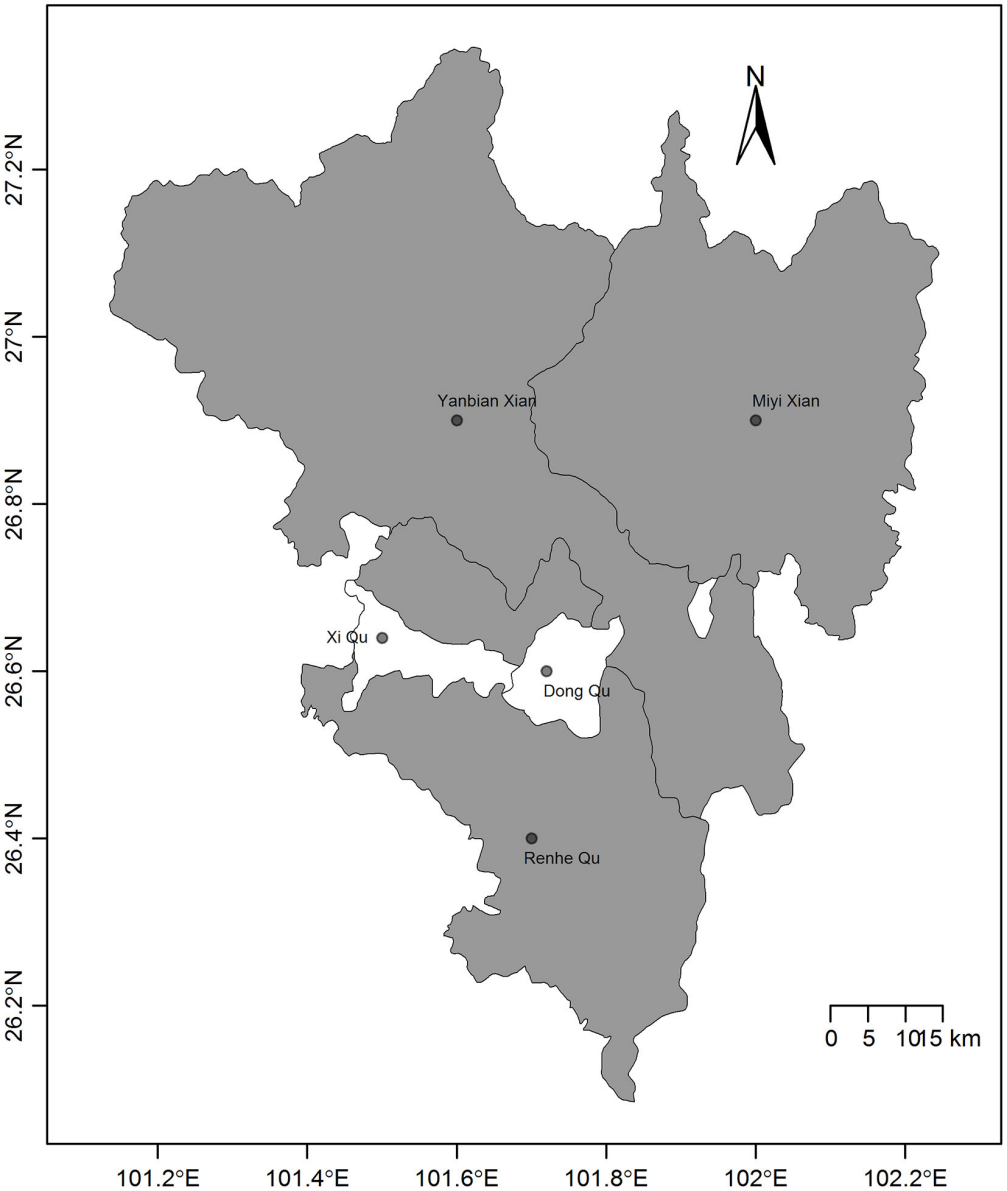
Map of the 5 counties in Panzhihua, Southwest China. The gray counties have a high incidence of scrub typhus and are rural areas whereas the white counties have a low incidence of scrub typhus and are urban areas.

Panzhihua is 7,401 square kilometers in size and is situated at north latitude 26°05'N to 27°21'N and east longitude 101°08'E to 102°15'E. Panzhihua had a registered population of over 1.105 million individuals at the end of 2016 according to the 2017 Sichuan statistical yearbook, including 0.5235 million people comprising the agricultural population. Panzhihua is located at the junction of Sichuan and Yunnan provinces in Southwest China and is the first city in the upper reaches of the Yangtze River, where the Jinsha and Yalong rivers meet. The district is comprised of complex and diverse landform types, 6 of which (flat dam, platform, high hills, low-middle mountains, middle mountains, and mountain plains) account for 88.38% of the total area. The climate is characterized as a stereoscopic climate with a baseband in the south subtropical zone; the most prominent characteristic is the distinction between the dry and rainy seasons. Summers are rainy with high temperatures and a relatively high humidity index. Winters are dry and sunny with a higher temperature compared with other areas, which can also be indicated from the annual mean temperature ranging from 18 to 25°C. There are 5 counties in Panzhihua (Figure 1).

### Data Collection

#### Surveillance Data of the Disease

We extracted the computerized dataset on notified scrub typhus cases in rural areas of Panzhihua from the period of January 1, 2008 to December 31, 2017 from the China Information System for Disease Control and Prevention. In China, all scrub typhus cases are diagnosed in terms of the uniform diagnostic criteria described by the Chinese Ministry of Health. Scrub typhus is diagnosed when a sick person displays at least three of the following criteria: a history (traveling to an endemic area and contact with chigger mites within 3 weeks before the onset of illness); sudden-onset high fever at the presence of characteristic eschar or ulcer; skin rash and lymphadenopathy; an agglutination titer >1:160 according to the Weil–Felix test (WF) using the OXK strain of *Proteus mirabilis*; and a 4-fold or larger increase in titres against *O. tsutsugamushi* in the indirect immunofluorescence antibody assay (IFA) (14). Scrub typhus has been a notifiable disease in Sichuan since 2006, similar to other provinces in China (13, 15). Since the notifiable network covers all hospitals and community health centers in Panzhihua, routine case reporting is performed by hospitals or community health centers through the National Notifiable Disease Report System (NNDRS) within 24 h. The notification system records detailed information of each case including birth day, gender, address, the onset date, diagnosis date, notification date, etc. The onset date of scrub typhus (15), which is more useful for epidemiological studies than the dates of diagnosis or notification dates, was used in our study. Basic population data for Panzhihua from 2008 to 2017 were obtained from Sichuan Statistical Yearbooks.

#### Meteorological Data

Meteorological data were obtained from the publicly available Chinese Meteorological Data Sharing Service System (CMDSSS) (16). Eight meteorological data variables were extracted from the CMDSSS during January 2008 to December 2017 for analysis: rainfall, sunshine, mean relative humidity, mean air temperature, mean land surface temperature, mean wind velocity, mean evaporation, and mean air pressure.

### Statistical Analysis

After obtaining a descriptive summary of each variable, Spearman's correlation analyses were performed to determine the correlations between meteorological variables. Then, the main study used DLNM (17) to describe the lag non-linear effects between meteorological factors and scrub typhus assuming that the incidence obeyed the Poisson distribution with overdispersion. Furthermore, because weekly scrub typhus cases are commonly sporadic, generalized additive models was used following a quasi-Poisson family (18).

First, the model was constructed for scrub typhus cases according to the basic idea of DLNM. After reviewing a significant amount of relevant research, exploring the cross-basis function was considered for meteorological variables including rainfall, sunshine, relative humidity, air temperature and land surface temperature, which were deemed to have a close relationship with the incidence of scrub typhus (4, 13), to describe effects that vary simultaneously both along the space of these weather variables and in the lag dimension of their occurrence. A natural cubic spline (ns) function was used for the non-linear effect and the lag effect of selected meteorological variables (11, 19, 20). The degrees of freedom (df) (knots) and lag were chosen by the Akaike Information Criterion for quasi-Poisson models (QAIC) (17). When the df of both were 3, the smallest QAIC would be acquired with the following settings completely determined. At this time, the maximum lag for rainfall was set as 14 weeks whereas the maximum lags for the other selected variables were set as 16 weeks. The definition of maximum lags was comprehensively considered according to those characteristics (e.g., times associated with the lifestyle of mites and the incubation period of the disease) and by consulting existing findings (11); however, this study was unique in using weeks as the unit of analysis for time rather than months when DLNM was applied to investigate the effects of weather factors on scrub typhus.

Second, the rest of the collected meteorological variables including mean wind velocity, mean evaporation, and mean air pressure may influence the selected five weather variables above and the incidence of scrub typhus; thus, they were more or less regarded as confounders and were investigated with a flexible modeling tool, using ns functions with an empirical 3 df (18) Consideration was mainly based on the direct interaction of meteorological variables, which was shown via correlation coefficients.

In addition, we did not simply and directly use year or month as variables controlling the variations of the long-term patterns and seasonality of the incidence of scrub typhus in the model. Because our aim was to explore whether short-term variation in scrub typhus cases was explained by exposure to weather factors, the long-term patterns including seasonality were controlled using an ns function with 3 df per year (21). Using week as a time analysis unit provided the advantage of reducing variation caused by the day of the week. After all the settings were determined, the smallest QAIC was 1424.573.

We included all meteorological variables and other potential factors including long-term and seasonal trends in the final model:


$$Y_{t}\sim quasipoisson\left(\mu_{t},\emptyset \mu_{t}\right)\;$$

$$E\left(y_{t}\right)=\mu_{t}Var\left(y_{t}\right)=\emptyset \mu_{t}\;$$

$$\begin{array}{l} \log\left(\mu_{t}\right)=\alpha +cb\left(P_{t,l}\right)+cb\left(G_{t,l}\right)+cb\left(T_{t,l}\right)+cb\left(R_{t,l}\right)+cb\left(S_{t,l}\right)\;\\ \;+ns\left(time,3^{*}year\right)+ns\left(W_{t},3\right)+ns\left(E_{t},3\right)+ns(F_{t},3)\; \end{array}$$

*Y*_*t*_ represents the weekly number of scrub typhus cases during week t; μ_*t*_ represents the expected number of weekly scrub typhus cases; ∅ is the dispersion parameter; and α is the intercept. *cb*() is a cross-basis function representing a bi-dimensional exposure-lag-response function for fitting the non-linear and lag effects of relevant weather factors, and *P*_*t, l*_, *G*_*t, l*_, *T*_*t, l*_, *R*_*t, l*_ *and S*_*t, l*_ in brackets represent weekly aggregate rainfall(RF), weekly mean land surface temperature(LST), weekly mean air temperature(AT), weekly mean relative humidity(RH), and weekly aggregate sunshine(SS), respectively. *ns*() represents ns function; time represents 1–522 weeks; *W*_*t*_, *E*_*t*_ *and F*_*t*_ represent weekly mean wind velocity(WV), weekly mean evaporation(ER), and weekly mean air pressure(AP), respectively.

The reference values were also defined for each weather variable before analyzing and estimating the relative risks (RR) which were calculated as well as the 95% confidence intervals for each weather variable when their exposure levels were 50, 60, 70, 75, 80, and 90% compared to the reference values. The reference value of weekly aggregate RF was 0 mm, and the 25th percentiles of the other weather variables were set as the reference values for weekly mean relative RH, weekly mean LST, weekly mean AT and weekly aggregate SS, which corresponded to 40.29%, 19.13°C, 16.90°C and 43.7 h, respectively. The effects of each meteorological factor on the incidence of scrub typhus were analyzed after different exposure levels.

All the implementations above were accomplished with R-3.5.0 using the dlnm (22) and mgcv packages.

## Results

### Characteristics of Scrub Typhus Cases

During the period of 2008–2017, 1,758 scrub typhus cases were reported in Panzhihua, among which 1,731 cases occurred in the countryside. The annual average incidence in rural areas was 25.77 per 100,000, ranging from 12.38 to 35.02 per 100,000. The highest incidence occurred from 23th to 44th weeks of each year and accounted for 94.74% of the entire year; a single epidemic peak occurred in the summer each year. Of the rural cases, 48.53% (840/1,731) occurred in males and 51.47% (891/1,731) occurred in females, corresponding to a male-to-female ratio of 0.94:1. The largest proportion of patients were in the 18–59-year-old age group (the young and middle-aged population), which accounted for 56.7% (981/1,731) of rural cases. According to occupation, 68.11% (1,179/1,731) of cases occurred in farmers, whereas elementary and nursery children and students accounted for 14.67% (254/1,731) and 12.02% (208/1,731) of cases, respectively.

### Characteristics of Meteorological Factors

**Figure 2:**
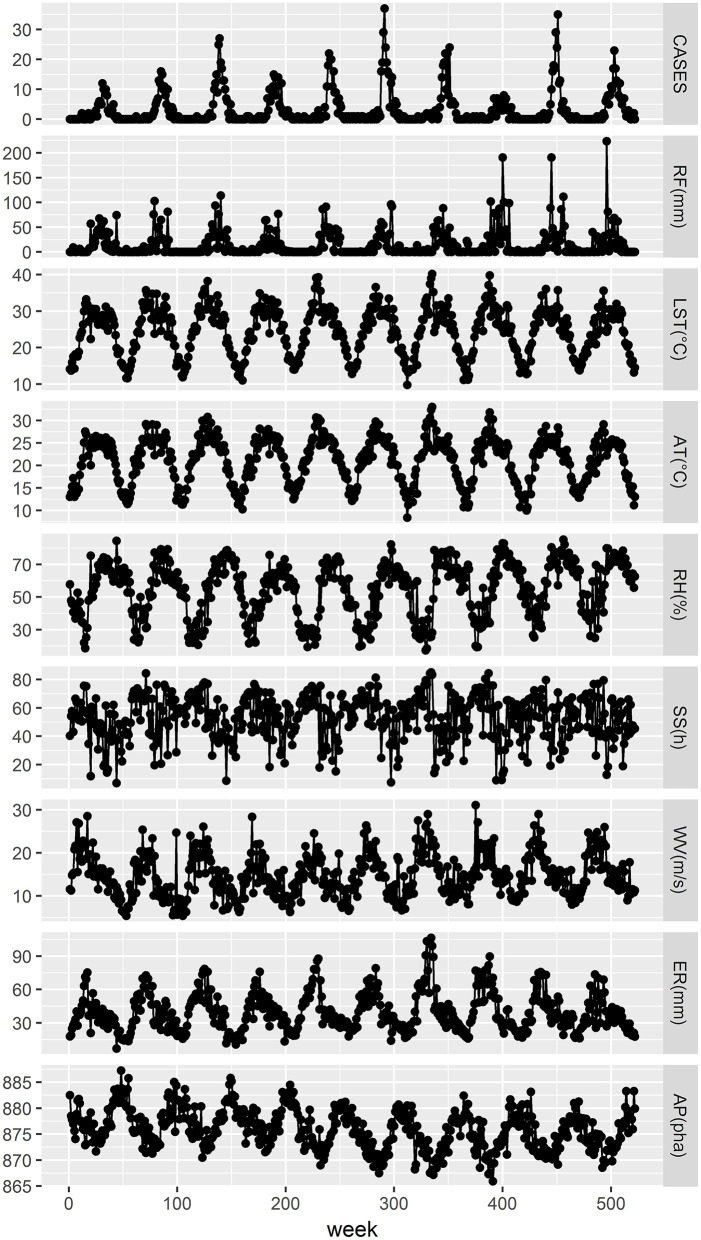
Weekly time series of the number of scrub typhus cases and meteorological variables in rural areas of Panzhihua district, Southwest China, 2008–2017. RF, LST, AT, RH, SS, WV, ER, and AP represent the weekly values of aggregate rainfall, mean land surface temperature, mean air temperature, mean relative humidity, aggregate sunshine, mean wind velocity, mean evaporation, and mean air pressure, respectively.

The weekly aggregate RF ranged from 0 to 223.50 mm, with a median of 1.45 mm. The weekly aggregate SS ranged from 7 to 85.20 h, with a median of 55.8 h. The weekly minimum and maximum mean LSTs were 9.83 and 40.19°C, respectively, with an average of 24.54°C. The weekly minimum and maximum ATs were 8.41 and 33.06°C, respectively, with an average of 21.18°C. The weekly mean relative RH ranged from 17.43 to 85.29%, with an average of 54.64%. The weekly mean WV ranged from 0.54 to 3.11 m/s, with an average of 1.48 m/s. The weekly mean ER ranged from 7 to 106.57 mm, with an average of 39.58 mm. The weekly mean air pressure (AP) ranged from 865.94 to 887.26 hPa, with an average of 875.90 hPa. Figure 2, Table 1 show the weekly time series for the number of cases and meteorological information during the investigation period. We can conclude from Figure 2 that before the number of scrub typhus cases increased significantly, rainfall, relative humidity, land surface temperature and air temperature displayed obvious fluctuations, and some time after they reached their peak, the number of scrub typhus cases peaked.

**Table 1:** Summary statistics for weekly scrub typhus cases and meteorological variables in rural areas of Panzhihua district, Southwest China, 2008–2017.

	**Total**	**Mean**	**S.D**.	**Min**	**P (25)**	**Median**	**P (75)**	**Max**
Aggregate rainfall (mm)	-	14.75	26.88	0	0	1.45	17.88	223.50
Aggregate sunshine (h)	–	53.29	15.94	7.00	43.70	55.80	65.40	85.20
Mean relative humidity (%)	–	54.64	17.02	17.43	40.29	59.00	68.39	85.29
Mean land surface temperature (°C)	–	24.54	6.56	9.83	19.13	25.49	29.65	40.19
Mean air temperature (°C)	–	21.18	5.13	8.41	16.90	22.24	25.20	33.06
Mean wind velocity (m/s)	–	1.48	0.51	0.54	1.09	1.42	1.83	3.11
Mean evaporation (mm)	–	39.58	17.73	7.00	26.61	36.00	49.86	106.57
Mean air pressure (hpa)	–	875.90	3.89	865.94	872.90	875.81	878.50	887.26
No. of cases of scrub typhus	1731	3.32	5.83	0	0	1	4	37

*Note: all the data are presented as the weekly aggregate or mean values*.

*S.D. = Standard deviation*.

As shown in Table 2, the weekly rainfall was positively correlated with relative humidity, land surface temperature and air temperature (*p* < 0.001) and was negatively correlated with sunshine and air pressure (*p* < 0.001). The weekly relative humidity was negatively correlated with sunshine, wind velocity and evaporation (*p* < 0.001).

**Table 2:** Spearman correlation coefficients between weekly meteorological variables in rural areas of Panzhihua district, Southwest China, 2008–2017.

	**Rainfall**	**Relative humidity**	**Land surface temperature**	**Air temperature**	**Sunshine**	**Wind velocity**	**Evaporation**
Relative humidity	0.63^a^	1					
Land surface temperature	0.41^a^	−0.06	1				
Air temperature	0.46^a^	−0.03	0.98^a^	1			
Sunshine	−0.54^a^	−0.72^a^	0.26^a^	0.21^a^	1		
Wind velocity	0.07	−0.48^a^	0.45^a^	0.44^a^	0.25^a^	1	
Evaporation	0.05	−0.56^a^	0.78^a^	0.76^a^	0.56^a^	0.75^a^	1
Air pressure	−0.27^a^	0.2^a^	−0.66^a^	−0.71^a^	−0.28^a^	−0.55^a^	−0.68^a^

a *p < 0.001*.

### Relationship Between Meteorological Factors and Scrub Typhus Cases

**Figure 3:**
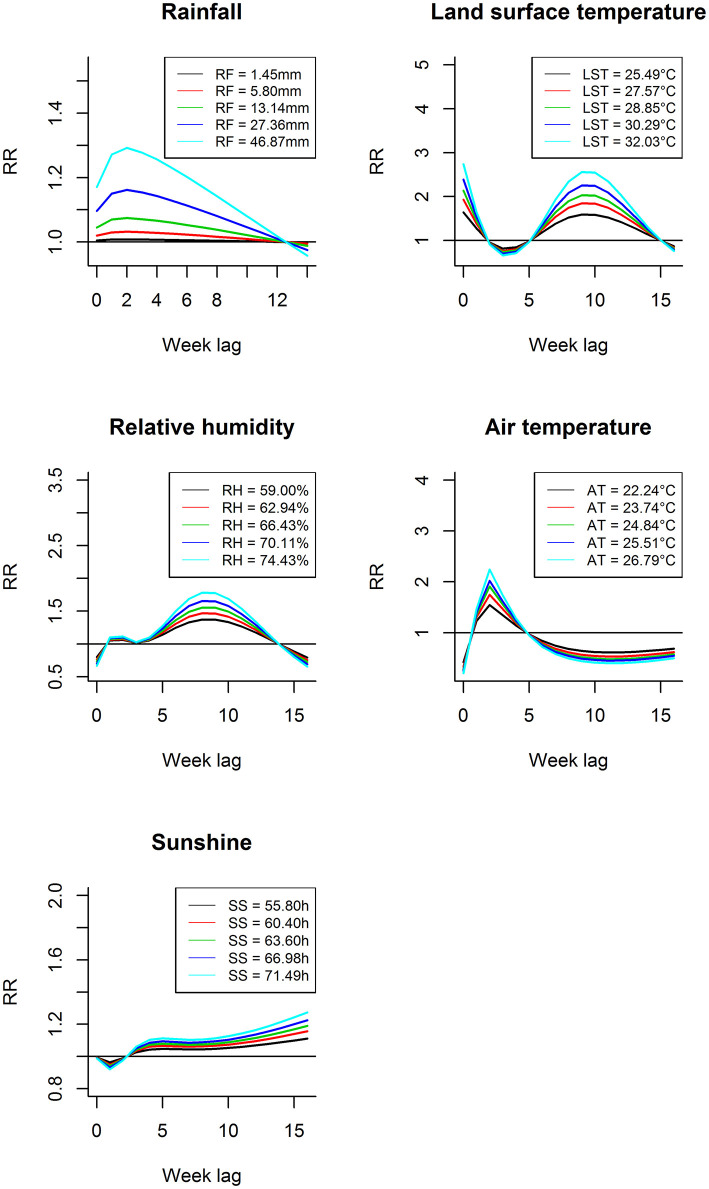
Lag-response curves for different weather variables in their diverse exposure levels (50, 60, 70, 80, and 90%).

Figure 3 shows the lag-response curves for how different weather variables affect the incidence of scrub typhus at different exposure levels (50, 60, 70, 80, and 90%) according to the model. There were obvious lagged associations between some weather factors (rainfall, relative humidity, and air temperature) and scrub typhus with the same overall trend, an inverse-U shape. The lag effect of land surface temperature on scrub typhus decreased in the beginning; however, after a few weeks, the relationship became significantly positive and then weakened at different hypothetical exposure levels. In addition, at the same lag time, the RR increased with the increase of exposure value for all climate variables when presenting a positive association.

**Figure 4:**
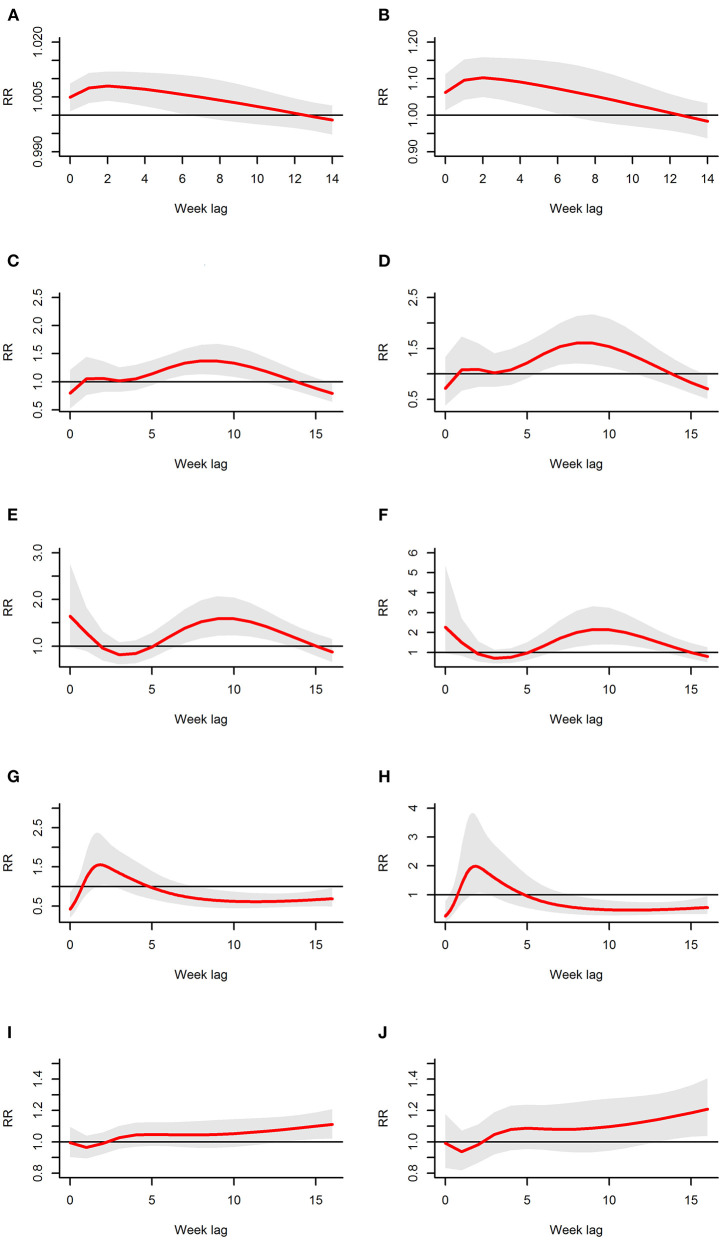
The estimates of distributed lag between meteorological variables and scrub typhus cases. The relative risk for each weather variable is calculated at 50 and 75% exposure levels The estimated distributed lag association is the red line, and the shaded bands indicate its 95% CI. **(A,B)** Show the scenario for rainfall; **(C,D)** show the scenario for relative humidity; **(E,F)** show the scenario for land surface temperature; **(G,H)** show the scenario for air temperature; and **(I,J)** show the scenario for sunshine.

Figures 4A,B showed some of the distributed lag associations between rainfall and scrub typhus cases. First, rainfall had a lag effect during the 0 to 12th weeks, but there was a significant correlation only during the 0 to 6th weeks; when there was less rainfall, the significant correlation range was reduced to ~0–5 weeks but peaked at the 2nd week. Second, when the rainfall was relatively low, the RR was low, and when the rainfall was relatively large, the RR was greater. Furthermore, the peak value of the RR increased as rainfall increased. For example, when the rainfall was 1.45 mm (50%), there was a significant correlation during the 0 to 5th weeks with the peak RR occurring at the 2nd week with an RR of 1.008 (95% CI: 1.004–1.012) (Figure 4A); when the rainfall was 17.88 mm (75%), there was a significant correlation during the 0 to 6th weeks with a peak RR also occurring at the 2nd week (RR: 1.103, 95% CI: 1.050–1.158) (Figure 4B).

Figures 4C,D showed some of the distributed lag associations between relative humidity and scrub typhus cases. First, the lag effect ranged from the first to 13th weeks with significance only during the 6th to 11th weeks, which lasted for~6 weeks and peaked at the 8th week. Second, similar to rainfall, there was a larger RR when the relative humidity was higher, and when the relative humidity increased, the peak RR also increased. For example, when the relative humidity was 59.00% (50%), there was a significant correlation during the 6th to 11th weeks with a peak RR occurring during the 8th week (RR: 1.372, 95% CI: 1.135–1.657) (Figure 4C). When the relative humidity was 68.39% (75%), a significant correlation also occurred during the 6th to 11th weeks with the peak RR occurring at the 8th week (RR: 1.608, 95% CI: 1.210–2.136) (Figure 4D).

Figures 4E,F showed some of the distributed lag associations between land surface temperature and scrub typhus cases. On the one hand, land surface temperature was associated with scrub typhus during the first 2 weeks and during the 6th to 15th weeks, but the significant correlation began at the 7th week and ended at the 13th week with a peak RR near the 9th week and lasting for 7 weeks. On the other hand, a larger RR value was associated with a higher land surface temperature; similarly, the peak RR was associated with the peak land surface temperature. When the land surface temperature was 25.49°C (50%), a significant association appeared during the 7th to 13th weeks with the peak value at the 9th week (RR: 1.589, 95% CI: 1.221–2.068), as shown in Figure 4E. When the land surface temperature was 29.65°C (75%), a significant association also appeared during the 7th to 13th weeks with the peak RR occurring at the 9th week (RR: 2.150, 95% CI: 1.391–3.322) (Figure 4F).

Figures 4G,H showed some of the distributed lag associations between air temperature and scrub typhus cases. Air temperature showed a similarly shorter and dramatic variation at the beginning of the lag time at different exposure levels; in other words, the zero week and the 8th to 16th weeks showed a statistically negative correlation, whereas the first to the 4th weeks displayed a positive risk peaking at the second week, which was the only statistically significant week. Regardless of whether there was a positive or negative risk, the RR and its corresponding peak value were larger in association with higher air temperatures. As a result, when the air temperature was 22.24°C (50%), a significant risk occurred at the second week, the only statistically significant positive week, with an RR of 1.546 (95% CI: 1.047–2.285), as shown in Figure 4G. A similar relationship occurred at 25.2°C (75%), with an RR of 1.970 (95% CI: 1.073–3.616), as shown in Figure 4H.

Figures 4I,J showed some of the distributed lag association between sunshine time and scrub typhus cases. At first, sunshine time displayed a negative correlation with no statistical significance during the 0–2nd weeks, and then it displayed a positive relative risk from the 3rd week with no ending time. Furthermore, the magnitude of the increasing trend became larger as sunshine time increased, and there was a significant positive increasing correlation during the 13th to 16th weeks with the largest value occurring at the 16th week. As a result, when the sunshine time was 55.80 h (50%), a significant risk appeared during the 13th to 16th weeks, with the increase of risk lasting the entire time; the largest RR value was 1.111 (95% CI: 1.021–1.209) at the 16th week, (Figure 4I); a similar association was displayed at 65.40 h (75%), with a largest RR of 1.208 (95% CI: 1.038–1.406) at the 16th week, as shown in Figure 4J.

## Discussion

Scrub typhus is a life-threatening vector-borne infectious disease that manifests as an acute indiscriminate febrile illness (23–25). Such infections are prevalent worldwide but are often undiagnosed/misdiagnosed, leading to a life-threatening condition (26–28). Meanwhile, no vaccine against *O. tsutsugamushi* is currently available (29). In survivors, immunity does not last long and is poorly cross-reactive amongst numerous strains. Hence, the disease deserves further scrutiny.

Our analysis results show that there is a high incidence of scrub typhus in rural areas in Panzhihua, Southwest China, similar to other regions of China (11, 15, 30, 31). To date, our results show that cases have been reported throughout the year due to the warmer environment.

Since weather factors such as temperature and humidity have been proven to have a significant relationship with the occurrence and transmission of many infectious diseases (9, 32–34), in our study, the association between weather factors and scrub typhus was explored using DLNM (17, 35).

Rainfall, temperature, humidity and other weather variables affect both the vectors and the agents they transmit in many ways (9).

First, certain trombiculid mite species transmit *O. tsutsugamushi*, a causative pathogen of scrub typhus, to humans via bite in the larval stage (12). The optimum temperature for growth, development and activity of the majority of chigger mites is 20–23°C; the growth rate will slow down until death in the presence of temperatures that are too high or too low (12).

Second, chigger mites are considered a class of arthropod vectors and reservoirs of *O. tsutsugamushi* (30). *O. tsutsugamushi* is a very small coccobacillus, an obligate intracellular parasite of infected mites, mammals and human beings (29); this species is also affected by temperature (12).

In summary, meteorological variables can affect scrub typhus cases both through their effects on the vectors and on the pathogens they transmit. In addition, a few studies have shown that there were no significant correlations between meteorological factors and monthly scrub typhus cases in Korea as a whole (8). Another study pointed out that because the seasonal distribution of scrub typhus varies in different geographical areas, it may be too simplistic to investigate the disease at a national level (15). Here, our work was conducted in rural areas of Panzhihua District, a subtropical zone, Southwest China, which may overcome this problem.

The results showed that different meteorological factors including supposedly diverse exposure levels have different patterns and magnitudes of lagged associations. First, rainfall is associated with scrub typhus with a delayed correlation and a relatively long lag range of 13 weeks with 7 weeks being statistically significant at all exposure levels. Second, the current study supports earlier studies from other areas in China (11) that demonstrate that vectors of scrub typhus are more abundant during the wet season.

One study revealed that chiggers survive and reproduce well at a relative humidity above 50% but decrease in number or activity when the relative humidity is below 50% (36); thus, when the assumed exposure level of the relative humidity increases, the RR of the lag association also increases.

Land surface temperature has a longer lag range of 12 weeks with 7 weeks being statistically significant and larger RRs for all assumed exposure levels. Some papers have indicated that land surface temperature affects the growth and development of the vector and pathogens it carries and also influences the abundance and distribution of rodents, which are major parasitic hosts for chigger mites (11). Our study indicates that a higher land surface temperature will introduce a larger RR and peak RR of the correlation compared with the reference level.

Air temperature has a shorter lag range of 4 weeks, but the ~1-week lag at the 2nd week is statistically significant for all assumed exposure levels. This time is shorter than that reported in previous studies (11, 15). As we know, the incubation period of scrub typhus is ~6–21 days (mean 10–12 days) after the initial chigger bite; thus, weather variables might not affect scrub typhus emergence immediately (15). We found that a higher temperature results in a greater relative risk at the same lag time from the results. These results coincide with studies conducted in a similar climate region (11).

In our study, sunshine time during the 0–2nd lag weeks was negatively correlated with weekly cases of scrub typhus with no significance, whereas sunshine time during the 3–16th lag weeks was positively correlated with the disease with the last 4 weeks being statistically significant. This can be explained by the abundance of chigger mites (37) and human outdoor activity. A longer sunshine time may have a protective effect for scrub typhus by inhibiting chigger activity, whereas in the long term, human outdoor activities may increase, and thus the occurrence of human infection may also increase.

To our knowledge, this study was the first to use week as a time analysis unit for the relationship between weather factors and scrub typhus rather than month; thus, a more precise lag range was obtained for the predetermined diverse exposure level. Consequently, the results have a greater public health significance for the prevention and control of scrub typhus. Furthermore, this was the first study to apply a mathematical model to analyse the relationship between weather variables and scrub typhus in this region. Additionally, only a few studies have analyzed the delayed effects of weather factors with a distributed lag non-linear model, and our study considered more different weather factors to simultaneously represent the exposure-response relationships and their temporal structure.

Some limitations of this study should be considered. First, there is inevitable underreporting in any infectious disease surveillance system together with less attention paid to scrub typhus; thus, areas of future improvement include training physicians regularly or improving diagnostics with the introduction of sensitive experimental equipment, particularly in rural regions. Second, the occurrence of scrub typhus is complex. It is indeed influenced by climate as shown by previous studies, but it is also influenced by other risk factors such as socioeconomic and behavioral risk factors (38). In addition, the pre-defined maximum lags for weather variables were used. The lag lengths were considered comprehensively according to existing studies (11, 15). These limitations should be evaluated in future studies.

## Conclusions

In summary, our study reveals that rainfall, sunshine, relative humidity, air temperature and land surface temperature have different patterns and magnitudes for the lagged correlation between weather factors and scrub typhus, and when all meteorological factors are at a high level, the potential risk of scrub typhus increases. The lag shape and association for meteorological information is applicable for developing an early warning system for scrub typhus. Public health professionals and medical service providers should pay more attention to preventing and controlling a potential increased risk of scrub typhus under the condition of high-level weather factors.

## Data Availability Statement

The raw data supporting the conclusions of this article will be made available by the authors, without undue reservation.

## Author Contributions

HL and YL: conceptualization. HL, XS, and SW: data curation. JH, FY, and BG: formal analysis. HL, JH, XS, and WW: methodology. YL: project administration and supervision. HL: resources, software, and writing—original draft. JH, XS, and YL: writing—review & editing. All authors contributed to the article and approved the submitted version.

## Funding

This work was supported by grants from the Health and Family Planning Commission Project of Sichuan Province in China (No. 18PJ576).

## Conflict of Interest

The authors declare that the research was conducted in the absence of any commercial or financial relationships that could be construed as a potential conflict of interest.

## Publisher's Note

All claims expressed in this article are solely those of the authors and do not necessarily represent those of their affiliated organizations, or those of the publisher, the editors and the reviewers. Any product that may be evaluated in this article, or claim that may be made by its manufacturer, is not guaranteed or endorsed by the publisher.
